# Reusable Surface-Modified Bacterial Cellulose Based on Atom Transfer Radical Polymerization Technology with Excellent Catalytic Properties

**DOI:** 10.3390/nano9101443

**Published:** 2019-10-11

**Authors:** Xin Li, Quan Feng, Dawei Li, Narh Christopher, Huizhen Ke, Qufu Wei

**Affiliations:** 1 Key Laboratory of Eco-Textiles, Ministry of Education, Jiangnan University, 1800 Lihu Avenue, Wuxi 214122, China; lx160401@163.com (X.L.); dawei1026@jiangnan.edu.cn (D.L.);; 2 Key Laboratory of Textile Fabric, Anhui Polytechnic University, Wuhu 241000, China; fengquan@ahpu.edu.cn; 3 Fujian Key Laboratory of Novel Functional Textile Fiber and Materials, Minjiang University, Fuzhou 350108, China

**Keywords:** gold nanoparticles, bacterial cellulose, ATRP, catalytic, reusability

## Abstract

The high catalytic activity of membrane-binding gold nanoparticles (AuNPs) makes its application in oxidation or reduction an attractive challenge. Herein, surface-functionalized bacterial cellulose (BC-poly(HEMA)) was successfully prepared with 2-hydroxyethyl methacrylate (HEMA) as monomers via the atom transfer radical polymerization (ATRP) method. BC-poly(HEMA) was further utilized as not only reducing agent but also carrier for uniform distribution of the AuNPs in the diameter of about 8 nm on the membrane surface during the synthesis stage. The synthesized AuNPs/BC-poly(HEMA) exhibited excellent catalytic activity and reusability for reducing 4-nitrophenol (4-NP) from NaBH_4_. The results proved that the catalytic performance of AuNPs/BC-poly(HEMA) was affected by the surrounding temperature and pH, and AuNPs/BC-poly(HEMA) maintained the extremely high catalytic activity of AuNPs/BC-poly(HEMA) even after 10 reuses. In addition, no 4-NP was detected in the degradation solution after being stored for 45 days. The reusable catalyst prepared by this work shows a potential industrial application prospect.

## 1. Introduction

Gold nanoparticles (AuNPs) are one of the most promising NPs compared with various metal and metal oxide NPs [1], owing to their excellent catalytic performance in a variety of chemical reactions including reductive catalysis of chlorinated [2], organic synthesis [3] and low-temperature CO oxidation [4]. However, AuNPs are prone to aggregation in reaction systems due to high surface energy, and then the catalytic activity is reduced [5]. In addition, AuNPs recycling is also a serious challenge [6]. Bioinorganic hybrid nanostructures can effectively combine the optical, electronic and mechanical properties of inorganic nanomaterial with good biocompatibility and low cost of natural biological materials, which have potential applications in optics [7], electronics [8], magnetism [9], catalysts [10], and battery materials [11]. Zawada et al. have reported a facile synthetic method of preparation of core-shell nanostructures using easy to obtain TEMPO (2,2,6,6-tetramethylpiperidine-1-oxyl)-coated gold nanoparticles as precursor [12]. Therefore, polymers can be used as the carriers for AuNPs due to its abundant active sites, thus obtaining reusable and stable AuNPs [13].

Recently, biocompatible degradable polymers have been used as AuNP carriers [14]. Zhang et al. reported a novel Au/Bi_12_O_17_C_l2_ composite for methyl orange and phenol degradation [15]. Nguyen et al. developed Au@g-C_3_N_4_ nanocatalysts in reducing nitroaromatic compounds [16]. However, most methods in the literature involve toxic or dangerous reductants. Cellulose, as the most abundant polymer on Earth [17], has been proved to be a green reducing agent for AuNPs synthesis [18]. Zhang et al. mesoporous titania networks were synthesized by using bacterial cellulose (BC) membranes as biotemplates [19]. Li et al. reported the preparation of metal nanoparticles with functional cellulose as reducing agent [20]. BC is suitable for all fields of application, due to its various advantages including green character, economical, biocompatibility, high ultrafine porosity, high mechanical strength and ease of chemical modification [21]. It was noticed that AuNPs effectively prevented agglomeration and promoted uniform dispersion in the unique network structure of BC [22]. On the other hand, BC can provide a channel for the mass transfer process in catalytic reactions to increase the contact rate with AuNPs, and improve the catalytic efficiency [23]. In particular, BC-loaded AuNPs membrane possess excellent reusability [24].

In the preparation of AuNPs from cellulose as a carrier, it is necessary to enhance the interaction between cellulose and AuNPs due to particle aggregation, but this remains a challenge [25]. As previous studies have shown, 2-hydroxyethyl methacrylate (HEMA) can be grafted onto cellulose fibers in the presence of ethylene [26]. Pure poly(HEMA) itself is a unique synthetic polymer used in biomedical fields, including enzyme immobilization [27], artificial cornea [28] and soft contact lens [29]. Hydroxyl (–OH) and acrylate groups on HEMA monomers are suitable sites for metal ions and harmful dyes to bind by chelation and ion-ion exchange [30]. Atom transfer radical polymerization (ATRP), one of the controlled/quasi-living radical polymerization techniques [31], has often been adopted for making linear polymer chains/brushes with controlled molecular lengths/weights since the propagation centers do not undergo chain termination and/or chain transfer during polymerization, and thus the molecular weights/lengths increase linearly with the conversion of monomers [32]. During the process of ATRP modification, bromine derivatives were applied as the initiators, because, the bond between the polymer chain terminated by HEMA and chlorine is too strong; thus the migration of the terminal group between the metal complex and active polymer chain is very slow [33], By applying bromine derivatives as the initiators, significantly better results might be obtained in comparison with chlorine derivatives [34].

In this study, BC was firstly modified by ATRP, and then BC-poly(HEMA) was used as reducing agent and stabilizer, and the catalytic activity and reusability of AuNPs/BC-poly(HEMA) were investigated. This study also explored the effects of temperature and pH on catalytic reduction. Additionally, the reusability of the AuNPs/BC-poly(HEMA) was demonstrated by reusing it 10 times. The results indicated that the AuNPs/BC-poly(HEMA) was an efficient reusable catalyst.

## 2. Experiment

### 2.1. Materials

BC was prepared in-house (Jiangnan University, Wuxi, China). Hydrochloroauric acid trihydrate(HAuCl_4_·3H_2_O, 99.9%), NaBH_4_ (≥97%) were supplied by Sinopharm Group Chemical Reagent Co. Ltd. (Shanghai, China). N,N-dimethylformamide (DMF), tetrahydrofuran (THF), 4-nitrophenol (4-NP, ≥99%), CuCl, 2-bromoisobutyryl bromide (2-BIB), triethylamine (TEA), 1,1,4,7,10,10-hexamethyltriethyl-enetetramine (HMTETA), 2-hydroxyethyl methacrylate (HEMA) were purchased from Aladdin Chemical Reagent Co. Ltd. (Shanghai, China) and used without further purification. All chemicals were analytical grade and were prepared with deionized water (DIW).

### 2.2. Preparation of Bacterial Cellulose 2-Hydroxyethyl Methacrylate (BC-Poly(HEMA))

BC was synthesized similar with a previous report [35].

#### 2.2.1. Initiation

BC membrane was immersed into THF (150 mL) for 20 min. Then the TEA (10 mM, 210 µL) and 2-BIB (10 mM, 189 µL) were added into THF for 3.5 h at 35 °C. The prepared BC-Br initiator was extracted and stored in THF.

#### 2.2.2. Surface Grafting

**Figure 1:**
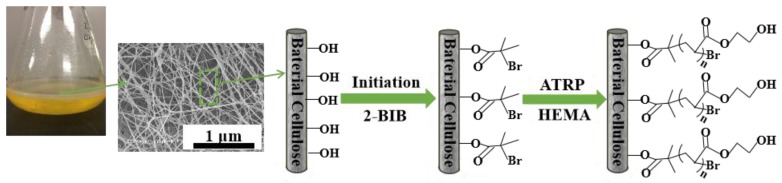
Schematic showing the grafting of poly 2-hydroxyethyl methacrylate (poly(HEMA)) on the surface of bacterial cellulose (BC) membrane via the atom transfer radical polymerization (ATRP) method.

Prior to this reaction, HEMA (24 mL) and a mixture of HMTETA (400 µL) and DMF (24 mL) were deoxygenated through three freeze-pump-thaw cycles by using liquid nitrogen, and CuCl (100 mg) was then added to the mixed system. After the HMTETA/DMF mixture is magnetically stirred in a vacuum glove box for 2 h, BC-Br and HEMA are placed into the mixture system. The reaction system was stored in the glove box at 25 °C, and the ATRP reaction of HEMA was completed in 6 h. Finally, BC modified by HEMA was washed by ethanol and dried in air. According to the empirical results, the graft rate of HEMA monomer onto the BC-poly(HEMA) membrane was 21.7 wt%. The detailed procedure for making BC-poly(HEMA) is schematically depicted in Figure 1.

### 2.3. Synthesis of Gold Nanoparticles (AuNPs)/BC-Poly(HEMA)

HAuCl_4_ (1.0 mM, 20 mL) aqueous solution in a 50 mL Erlenmeyer flask was heated to 110 °C. Then BC-poly(HEMA) was added to the boiling solution, and the reaction mixture gradually changed from light yellow to pink purple. Finally the membrane was taken out from the heated aqueous solution after 10 min, and afterwards washing it with DI water.

### 2.4. Catalytic Reduction of 4-Nitrophenol (4-NP)

**Figure 2:**
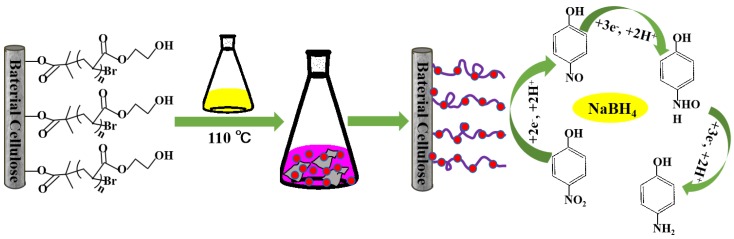
Illustration of the reduction mechanism of 4-nitrophenol (4-NP) by NaBH_4_ occurred on the surface of gold nanoparticles (AuNPs)/bacterial cellulose 2-hydroxyethyl methacrylate (BC-poly(HEMA)).

The catalytic activity of AuNPs/BC-poly(HEMA) was examined by a model reaction of reducing 4-nitrophenol (4-NP) from NaBH_4_. Typically, 50 mL mixed aqueous solution of 4-NP (0.5 mM) and NaBH_4_ (0.25 M) were added into a reagent bottle, then 5 mg AuNPs/BC-poly(HEMA) was placed into the well-mixing solution. As the reaction proceeded, the color of the mixed solution gradually changed from yellow to colorless and the reduction process was monitored by ultraviolet (UV)–visible spectrophotometer (U4100, Hitachi, Tokyo, Japan). The reduction mechanism of 4-NP by NaBH_4_ occurred on the surface of AuNPs/BC-poly(HEMA) was shown in Figure 2.

### 2.5. Materials Characterization

Surface morphologies of the BC, BC-poly(HEMA) were examined by a field emission scanning electron microscope (FE-SEM, Hitachi S4800, Tokyo, Japan). A transmission electron microscope (TEM, JEOL/JEM-2100, Hitachi, Tokyo, Japan) was used to analyze the structure and morphology of AuNPs, AuNPs/BC and AuNPs/BC-poly(HEMA) at the acceleration voltage of 100 kV. Fourier transform infrared spectroscopy (FT–IR, Nicolet Nexus, Thermo Electron Corporation, Waltham, MA, USA) was used to test the chemical structure of BC, BC-Br, BC-poly(HEMA). Using TGA (TGA-Q500, TA Instruments Corporation, New Castle, DE, USA) the thermal stability of samples were determined with the heating rate at 10 °C/min within the scope 30 to 800 °C under N_2_. The crystal structure of BC, AuNPs/BC-poly(HEMA) were measured by Powder D8 Advance X-ray diffraction (XRD, Bruker AXS D8, Leipzig, Germany). X-ray photoelectron spectroscopy (XPS, Escalab 250Xi, Thermo Scientific Escalab, Thermo Fisher Scientific, Waltham, MA, USA) was also used to perform the elemental analysis of BC-Br and AuNPs/BC-poly(HEMA). The optical spectra and catalytic activity of the AuNPs/BC-poly(HEMA) was examined towards a Hitachi U4100 UV-visible spectrophotometer.

## 3. Results and Discussion

### 3.1. Morphology Analysis

**Figure 3:**
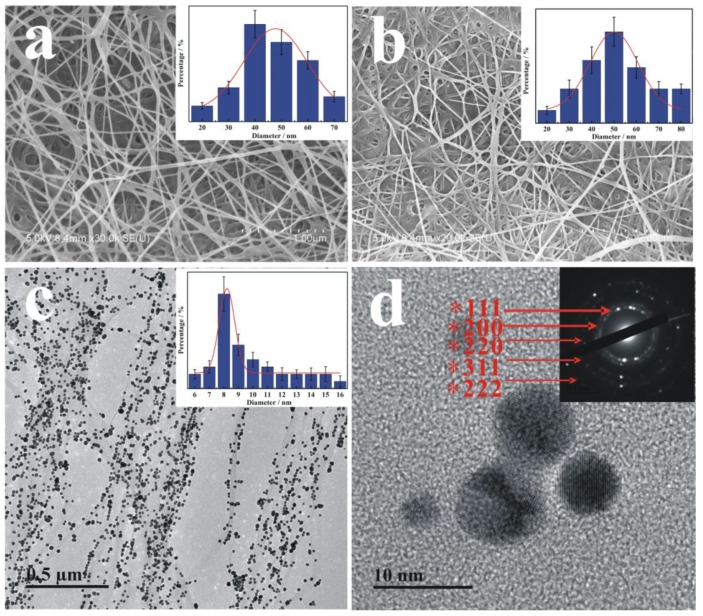
Scanning electron microscope (SEM) images of (**a**) BC and (**b**) BC-poly(HEMA) with the grafting rate of 21.7 wt% (inset image is a diameter of fibers of these materials in the case of BC-and BC-poly(HEMA)); transmission electron microscope (TEM) images of (**c**) AuNPs/BC-poly(HEMA), (**d**) as-synthesized AuNPs (enlarged), with the top right of inset presented selected area electron diffraction (SAED) pattern of AuNPs (high-resolution TEM (HR-TEM)).

The morphologies are shown in Figure 3 as carried out by FE-SEM and TEM. Figure 3a shows that the micro-pore structure of pure BC membrane with ultrafine nanofiber structure and non-woven mechanically robust network, which provided high specific surface area and porosity. In addition, the pure BC presents a typical 3D network structure. The size distribution range of BC (inset image of Figure 3a) was 20 to 70 nm, with an average diameter of about 45 nm. After surface modification by ATRP, BC-poly(HEMA) (Figure 3b) showed no obvious damage, and still maintained similar morphology to that of BC. This is due to the fact that the surface modification of ATRP caused little damage to the BC membrane under non-violent conditions; in addition, BC can withstand a certain impact because of its excellent mechanical properties. In the one-step method synthesis of AuNPs, the characteristic peaks were observed at 524 nm (Figure S1), which is consistent with the literature. Figure 3c depicts the successful formation of AuNPs/BC-poly(HEMA) with the average dimension of about 8 nm for AuNP, indicating that the BC-poly(HEMA) can act as the reducing agent in the preparation of gold nanoparticles. Additionally, the TEM images of pure BC and AuNPs/BC were depicted in Figures S2 and S3. The high-resolution TEM (HRTEM) image (Figure 3d) clearly shows the selected area electron diffraction (SAED) pattern of AuNPs, the diffraction ring of face-centered cubic structure of AuNPs was indexed as (222), (311), (220), (200) and (111) planes from outer to inner.

### 3.2. Characterization of BC-Poly(HEMA) 

**Figure 4:**
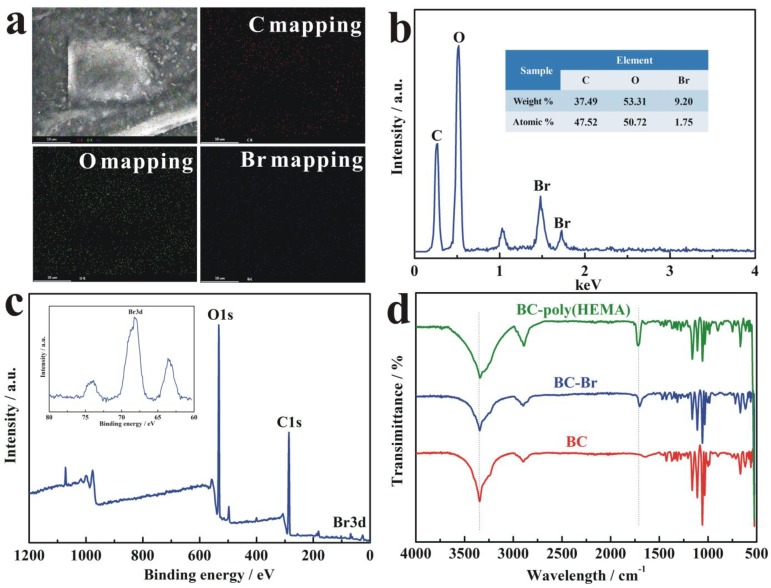
(**a**,**b**) Scanning electron microscopy–energy-dispersive X-ray spectroscopy (SEM–EDS) elemental mapping images of BC-Br; (**c**) X-ray photoelectron spectroscopy (XPS) spectrum of high-resolution C/O/Br on BC-Br; (**d**) FT-IR spectrum of BC, BC-Br, BC-poly(HEMA).

Figure 4a reveals that the SEM–energy-dispersive X-ray spectroscopy (EDS) mapping images contained C, O and Br elements on the surface of BC-Br initiator, thus indicating their uniform distribution in the BC membrane. Additionally, Figure 4b shows the observation of C, O and Br peaks on EDS spectrum, and the relative concentration of the above elements were about 37.49, 53.31 and 9.20 wt%, respectively. Furthermore, XPS of the BC-Br membrane are shown in Figure 4c, with the inset shown the magnified images at the binding energy range from 80 to 60 eV. The survey spectrum indicated the presence of C, O and Br elements, where the large peaks observed at 533 and 282 eV correspond to O1s and C1s, respectively. The high-resolution spectrum of Br 3d located at 68 eV confirms the binding of the Br-initiator to the BC membrane. Figure 4d depicts FT–IR spectra at the wavenumber from 4000 to 500 cm^−1^ acquired from BC, BC-Br and BC-poly(HEMA) to evaluate the effect of ATRP modification. As shown in Figure 4d, BC membrane has two characteristic bands centered at the wavenumber of 3347 cm^-1^ and 2905 cm^−1^, which were attributed to O–H stretching and C–H stretching, respectively. After initiation by 2-BIB, –H on the hydroxyl group was replaced by –Br, as the evidenced of the significant reduction of O–H stretching at the wavenumber of 3347 cm^−1^, and a new absorption band at the wavenumber of 1720 cm^-1^ was attributed to C=O stretching. By further modification of ATRP, the two FT–IR bands centered at 3347 cm^-1^ (O–H) and 1720 cm^−1^ (C=O) were obviously increased, due to the successful surface-grafting with HEMA.

### 3.3. Characterization of AuNPs/BC-Poly(HEMA)

**Figure 5:**
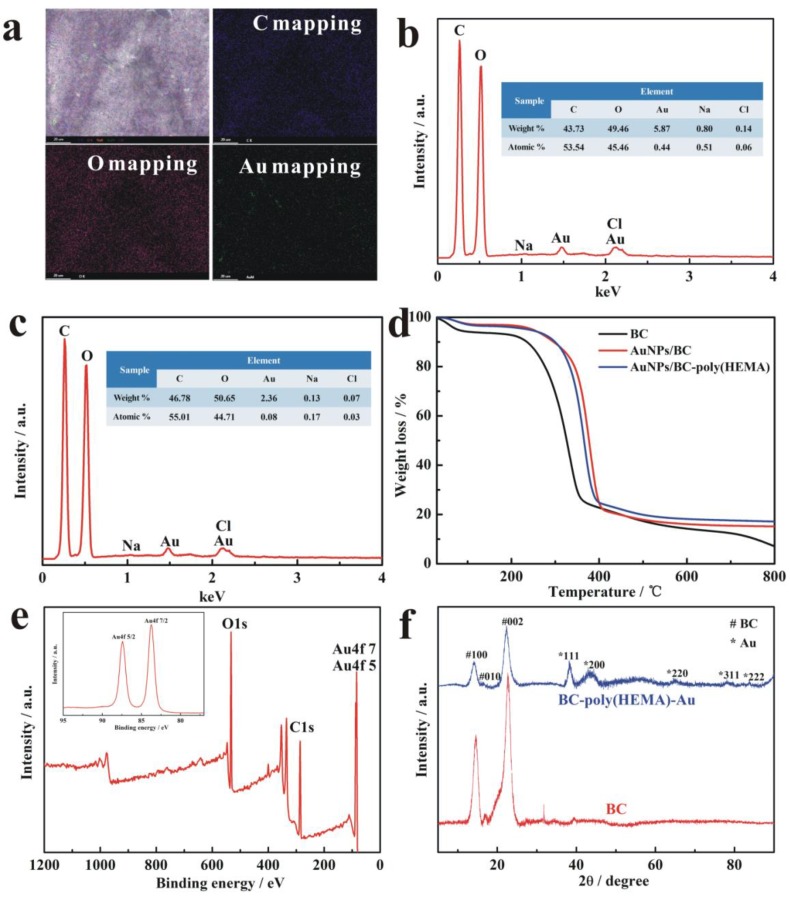
SEM-EDS elemental mapping images of AuNPs/BC-poly(HEMA) (**a**,**b**) and AuNPs/BC (**c**); (**d**) TGA curves of BC, AuNPs/BC and AuNPs/BC-poly(HEMA); (**e**) XPS spectrum of high-resolution C/O/Au on AuNPs/BC-poly(HEMA); (**f**) X-ray diffraction (XRD) patterns of AuNPs/BC-poly(HEMA).

SEM–EDS mapping images of AuNPs/BC-poly(HEMA) contained C, O and Au elements were verified as revealed in Figure 5a, indicated the uniform distribution of Au on the suface of membrane. Moreover, it can be seen that the relative concentration of C, O, Au, Na and Cl elements on EDS spectra in Figure 5b were about 43.73, 49.46, 5.87, 0.80 and 0.14 wt%, respectively, while the corresponding element content of AuNPs/BC were about 46.78, 50.65, 2.36, 0.13 and 0.07 wt% (Figure 5c), which indicated that the BC-poly(HEMA) had higher loading of AuNPs than pure BC.

TGA was used to study the thermal stability of BC, AuNPs/BC, AuNPs/BC-poly(HEMA).

The results shown in Figure 5d depicts three stage of weight loss. The first stage from ambient to 100 °C corresponds to the evaporation of water, while the other two stages of 100 °C to 350 °C and 350 °C to 600 °C are due to the collapse of a cellulose skeleton [36,37]. The total weight loss rates of BC, AuNPs/BC, AuNPs/BC-poly(HEMA) were 93 wt%, 86 wt% and 82 wt%, respectively, which indicated the improvement of thermal stability by protection from AuNPs. Meanwhile, Au content in the samples of AuNPs/BC and AuNPs/BC-poly(HEMA) determined on the basis of TGA were 7% and 11%, respectively, which indicated the higher loading of AuNPs on AuNPs/BC-poly(HEMA).

In addition, the Au oxidation state in the as-prepared AuNPs/BC-poly(HEMA) was obtained by XPS analysis as indicated in Figure 5c [38], with the inset showing the magnified images at the binding energy range from 95 to 77 eV. It can be seen that the strong peaks at 533 and 284 eV were attributed to the BC, and the fitted Au 4f peaks consisting of Au 4f 5/2 and Au 4f 7/2 distinctively showed on the magnified images at peaks of 87.4 and 83.7 eV, respectively. The results are in accordance with the literature values reported for Au (0) [39]. Au(III) peaks at 86.9 and 90.6 eV are absent in the XPS spectrum of the AuNPs/BC-poly(HEMA), which could be ascribed to the small amount of Au(III) in the reaction system after the hydrothermal reaction and confirmed the almost completed reduction of Au(III) to Au(0). Additionally, the XPS atomic percent values of Au atoms on AuNPs/BC-poly(HEMA) were 7.3%, while Au atoms on AuNPs/BC were only 3.1%.

Figure 5d shows the XRD patterns of as-prepared BC and AuNPs/BC-poly(HEMA) from 10° to 90° (2θ). Several diffraction peaks centered at 2θ of 14.2°, 16.9° and 22.7° correspond to the (100), (010) and (002) of BC. On the XRD pattern of AuNPs/BC-poly(HEMA), several peaks located at 2θ of 38.2°, 44.1°, 65.0°, 78.1°, and 83.4° appear, corresponding to the diffraction of (111), (200), (220), (311), and (222) planes of Au, respectively [40], also consistent with previous SEAD pattern of Au. XRD results confirmed the successful synthesis of AuNPs. Meanwhile, this also indicated that the presence of AuNPs had no obvious effect on the crystallography of BC.

### 3.4. Catalytic Activity of AuNPs/BC-Poly(HEMA)

**Figure 6:**
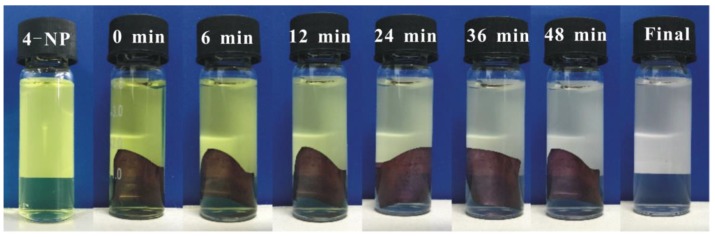
AuNPs/BC-poly(HEMA) in the 4-NP solution at different time intervals.

**Figure 7:**
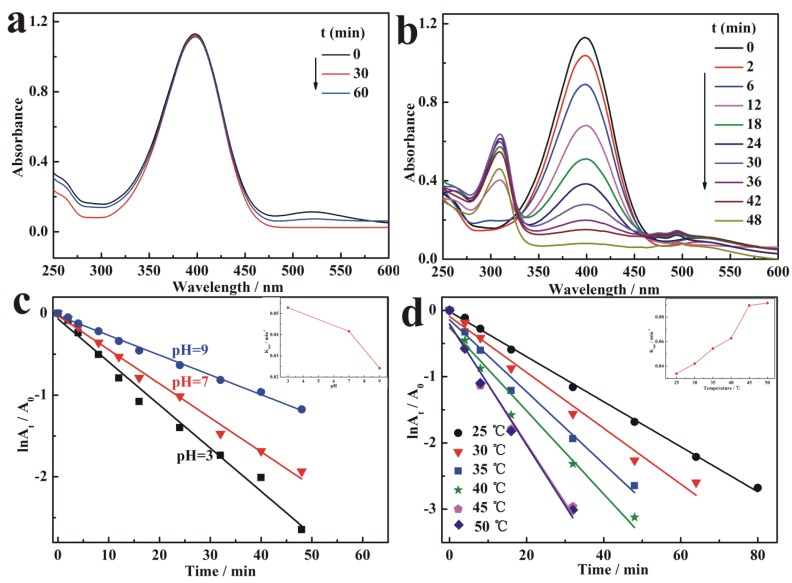
Time dependent ultraviolet–visible (UV−vis) spectra of the reduction of 4-NP at NaBH_4_ concentration of 0.25 M with (**a**) BC-poly(HEMA) and (**b**) AuNPs/BC-poly(HEMA), respectively; Plot of ln(A_t/_A_0_) versus time at (**c**) different pH (with inset showing the reaction rate constant k as a function of pH) and (**d**) different temperature (with inset showing the reaction rate constant k as a function of temperature).

As shown in Figure 6, the color of the reaction system faded from yellow to transparent. The reduction process was monitored by UV–visible spectroscopy. The yellow color of 4-NP solution changed deeper while added NaBH_4_, resulting in the maximum absorption peak of mixed solution transferred from 317 nm to 400 nm due to the formation of 4-nitrophenolate ion. Figure 7a shows the UV spectrum of 4-NP/NaBH_4_ with BC-poly(HEMA) and no obvious changes in the absorbance at 400 nm can be detected within 60 min. The results confirmed that BC-poly(HEMA) had almost no catalytic activity for the model reaction After replacing BC-poly(HEMA) with AuNPs/BC-poly(HEMA), the mixed aqueous solution showed a very different phenomenon with small bubbles continually released from AuNPs/BC-poly(HEMA), indicating the reduction reaction in the system. Figure 7b showed the absorbance intensity of the reaction system at 400 nm had a marked decline and a new peak presented at 300 nm on the typical time-dependent UV–vis spectra, which caused by the reduction of 4-NP to 4-AP. The reducing reaction was fast and the 4-NP can be almost completely reduced within 50 min.

The relationship between absorbance at 400 nm on the UV–vis spectrum and reaction time was plotted to reflect the reaction kinetics and estimate the catalytic activity of AuNPs/BC-poly(HEMA). Figure 7c displays the effect of pH on the catalytic activity of AuNPs/BC-poly(HEMA) toward the 4-NP reduction by using the model of the plot of ln(A_t/_A_0_) versus reaction time, where A_t_ is the absorbance at reaction time *t* and A_0_ is the initial absorbance. The inset image shows the apparent reaction rate constant k_app_ (min^−1^), which was obtained by fitting the experimental results. The result depicts that ln(A_t_/A_0_) decreases linearly with reaction time and the reduction of 4-NP by AuNPs/BC-poly(HEMA) was closely dependent on pH value in the system. As shown in the inset image of Figure 7c, the k_app_ for 4-NP reduction is 5.28 × 10^−2^, 4.16 × 10^−2^ and 2.42 × 10^−2^ min^−1^ at pH 3, 7 and 9, respectively, and it is evident that low pH is conducive to high reduction efficiency. The reduction of 4-NP over AuNPs/BC-poly(HEMA) in the presence of NaBH_4_ follows the Langmuir-Hinshelwood kinetics and the step of adsorption is the important part of reduction reaction [41]; therefore, the negatively charged BH^4−^ could be easily adsorbed on the surface of AuNPs/BC-poly(HEMA) at low pH values, resulting in an improvement of rate and efficiency for 4-NP reduction under acidic conditions.

Figure 7d shows the ln(A_t_/A_0_) versus reduction time at different temperatures to investigate the relationship between catalytic activity and temperature of AuNPs/BC-poly(HEMA). As shown in the inset image of Figure 7d, with the increase of temperature at the range of 25 °C to 50 °C the k_app_ for reduction reaction was in the range of 3.39 × 10^−2^ to 9.14 × 10^−2^ min^−1^. Meanwhile, k_app_ reached the highest value at 45 °C, and showed no obvious increase with the further increase of temperature. The heterogeneous catalytic reactions customarily obey the key molecular principle of catalysis, which corresponded to the Sabatier principle [42] stating that the substrate molecules must be adsorbed onto the catalyst and then activated, whilst the product molecules must also be desorbed. On the basis of this principle, the rate of catalytic reaction reaches a maximum when the rate of activation and product desorption were balanced. In this work, the balance was calculated at around 45 °C, where the maximum volcanic point was reached.

### 3.5. Reusable Stability of AuNPs/BC-Poly(HEMA)

**Figure 8:**
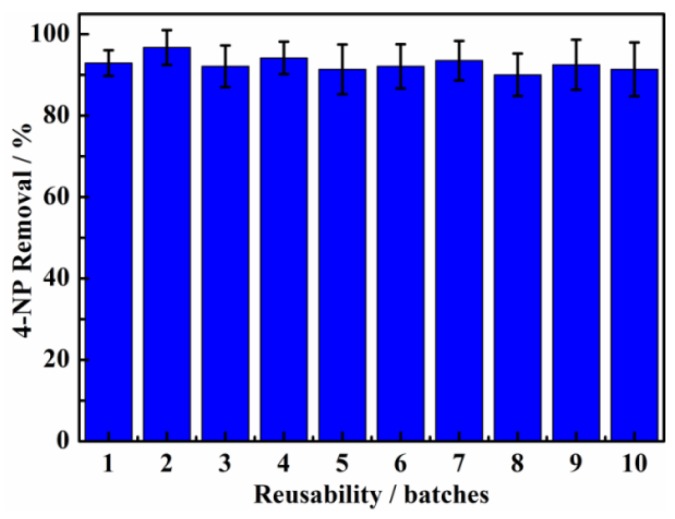
Reusability for the reduction of 4-NP catalyzed by the as-prepared AuNPs/BC-poly(HEMA).

The reusability must be considered in evaluating the engineering application of the catalyst. In this work, the reusability of AuNPs/BC-poly(HEMA) was examined through degradation of 4-NP for 10 cycles. As shown in Figure 8, the removal of 4-NP by AuNPs/BC-poly(HEMA) after reusing for 10 times was still around 90%, almost the same as the removal rate for the first use. Moreover, the TEM image (Figure S4) shows barely reduction in AuNPs on the surface of the AuNPs/BC-poly(HEMA) membrane after 10 reuses. Meanwhile, the comparison of catalytic activity by Au based nanocatalysts for the reduction of 4-NP was also shown in Table S1. These results clearly indicate that AuNPs/BC-poly(HEMA) is structurally stable with high catalysis efficiency and reusability. 

## 4. Conclusions

Herein, we developed a highly recyclable novel nanocatalyst of AuNPs/BC-poly(HEMA) for the reduction of 4-NP. The monomer of HEMA was successfully grafted on the surface of BC membrane via the ATRP method and then the BC-poly(HEMA) obtained acted as not only the reducing agent but also the carrier for preparing AuNPs. The experimental results showed that the as-prepared AuNPs/BC-poly(HEMA) was an excellent catalyst in the model reaction. The reduction efficiency and rate was highly dependent on environmental conditions, including pH and temperature. Moreover, the catalytic activity of AuNPs/BC-poly(HEMA) could be reused for 10 cycles without considerable loss. In addition, no 4-NP was detected in the degradation solution after being stored for 45 days, indicating that the AuNPs/BC-poly(HEMA) was a reliable green material and has a broad application prospect of catalytic transformation in the field of environmental remediation.

## Supplementary Materials

The following are available online at https://www.mdpi.com/2079-4991/9/10/1443/s1, Figure S1: UV-vis spectra of synthesized AuNPs, Figure S2: TEM image of pure BC, Figure S3: TEM image of AuNPs/BC, Figure S4: TEM image of Au NPs/BC-poly(HEMA) after 10 reuses, Table S1: Comparison of catalytic activity by Au based nanocatalysts for the reduction of 4-NP.
