# Supplementary material for: Reusable Surface-Modified Bacterial Cellulose Based on Atom Transfer Radical Polymerization Technology with Excellent Catalytic Properties
Source: Nanomaterials (Basel). 2019 Oct 11;9(10):1443. doi: 10.3390/nano9101443 (PMC6835580; doi:10.3390/nano9101443)
Supplement: Supplementary file 1 [file nanomaterials-09-01443-s001.pdf]

Supplementary Materials

Reusable Surface-Modified Bacterial Cellulose Based on Atom Transfer Radical Polymerization Technology with Excellent Catalytic Properties

Xin Li [1], Quan Feng [2], Dawei Li [1], Narh Christopher [1], Huizhen Ke [3,*] and Qufu Wei [1,3,*]

[1] Key Laboratory of Eco-Textiles, Ministry of Education, Jiangnan University, 1800 Lihu Avenue, Wuxi 214122, China; lx160401@163.com (X.L.); dawei1026@jiangnan.edu.cn (D.L.); doll6000000@yahoo.com (N.C.)

[2] Key Laboratory of Textile Fabric, Anhui Polytechnic University, Wuhu, Anhui 241000, China; fengquan@ahpu.edu.cn

[3] Fujian Key Laboratory of Novel Functional Textile Fiber and Materials, Minjiang University, Fuzhou, Fujian 350108, China

* Correspondence: kehuizhen2013@163.com (H.K.); qfwei@jiangnan.edu.cn (Q.W.)

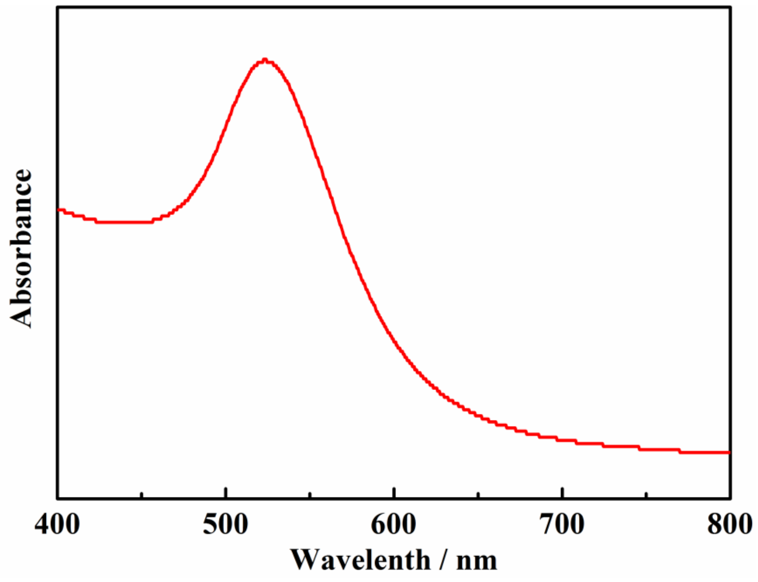

Figure S1. UV-vis spectra of synthesized AuNPs.

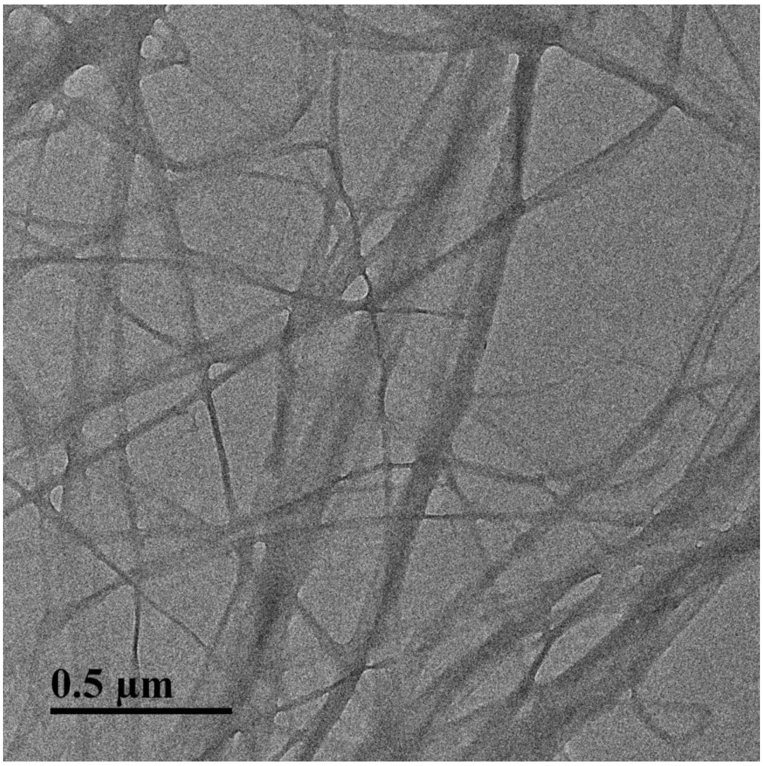

Figure S2 TEM image of pure BC

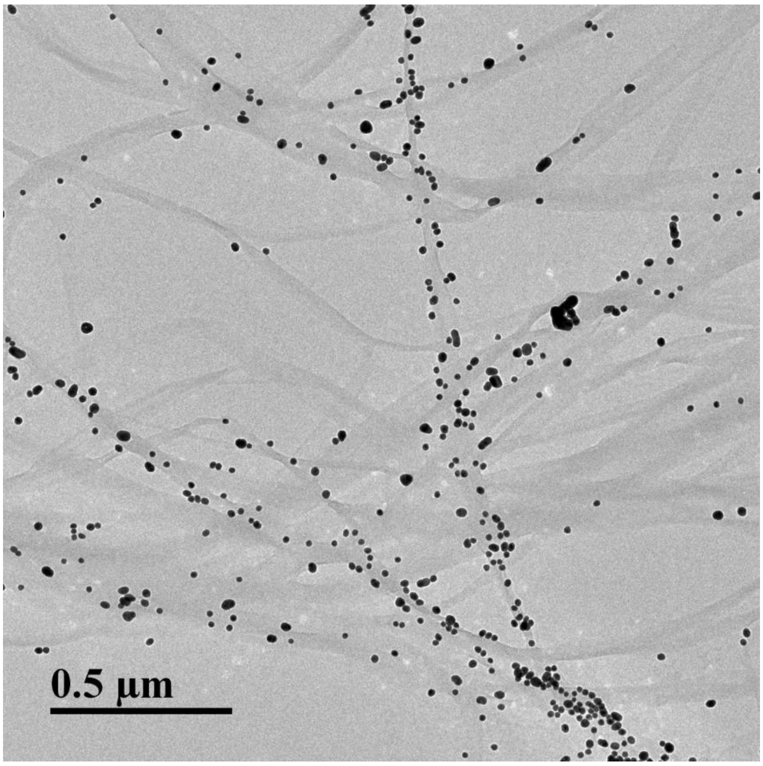

Figure S3 TEM image of AuNPs/BC

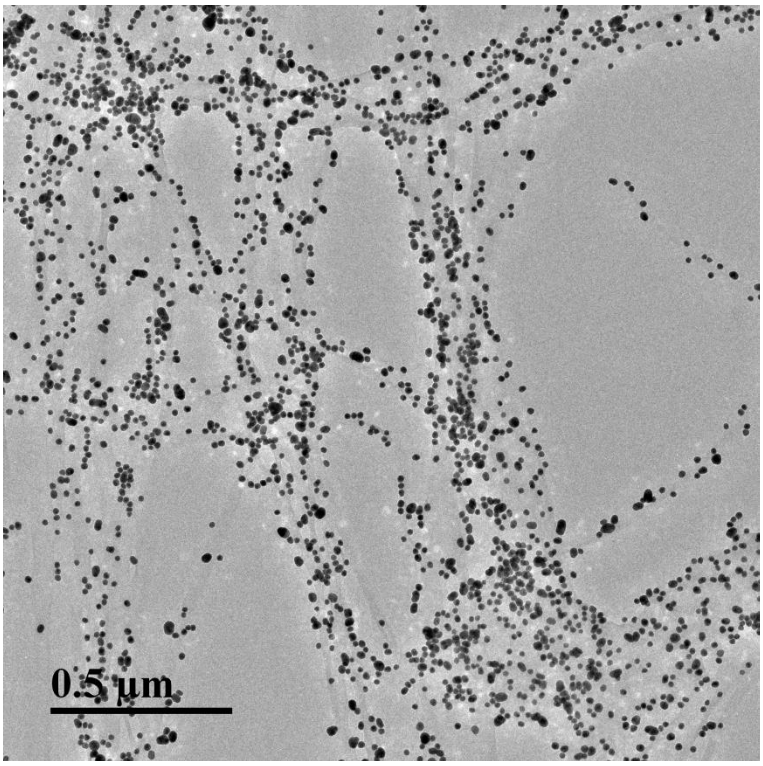

Figure S4 TEM image of Au NPs / BC-poly(HEMA) after 10 reuses

Table S1 Comparison of catalytic activity by Au based nanocatalysts for the reduction of 4-NP

Catalyst	4-NP (mM)	Size of Au (nm)	K (min^{-1})	Reusable time (batches)	Reference
Au/graphene hydrogel	0.093	15	1.9×10^{-1}	N/A	[1]
Polydopamine-Au	50	2.8	5.7×10^{-2}	10	[2]
Fe_3O_4@SiO_2-LBL-Au	0.16	4	3.4×10^{-1}	8	[3]
graphene/PDA-Au NPs	0.03	16	2.3×10^{-1}	N/A	[4]
AuNPs / BC-poly(HEMA)	50	8	9.14×10^{-2}	10	This work

Reference

[1] J. Li, C.Y. Liu, Y. Liu, Au/graphene hydrogel: Synthesis, characterization and its use for catalytic reduction of 4-nitrophenol, *J. Mater. Chem.* 22 (2012) 8426-8430.

[2] Y. Ni, G. Tong, W. Jie, H. Li, C. Feng, C. Yu, Y. Zhou, One-pot preparation of pomegranate-like polydopamine stabilized small gold nanoparticles with superior stability for recyclable nanocatalysts, *Rsc Adv.* 6 (2016) 40698-40705.

[3] Y. Zhu, J. Shen, K. Zhou, C. Cheng, X. Yang, C. Li, Multifunctional Magnetic Composite Microspheres with in Situ Growth Au Nanoparticles: A Highly Efficient Catalyst System, *J. Phys. Chem. C* 115 (2011) 1614-1619.

[4] J. Luo, N. Zhang, R. Liu, X. Liu, In situ green synthesis of Au nanoparticles onto polydopamine-functionalized graphene for catalytic reduction of nitrophenol, Rsc Advances 4 (2014) 64816-64824.
